# Mitigation of acetaminophen-induced liver toxicity by the novel phosphatidylinositol 3-kinase inhibitor alpelisib

**DOI:** 10.3389/fphar.2023.1212771

**Published:** 2023-08-07

**Authors:** Mohamed E. Shaker, Hesham A. M. Gomaa, Sara H. Hazem, Mohamed A. Abdelgawad, Mohamed El-Mesery, Ahmed A. Shaaban

**Affiliations:** ^1^ Department of Pharmacology, College of Pharmacy, Jouf University, Sakaka, Al-Jouf, Saudi Arabia; ^2^ Department of Pharmacology and Toxicology, Faculty of Pharmacy, Mansoura University, Mansoura, Egypt; ^3^ Department of Pharmaceutical Chemistry, College of Pharmacy, Jouf University, Sakaka, Al-Jawf, Saudi Arabia; ^4^ Department of Biochemistry, Faculty of Pharmacy, Mansoura University, Mansoura, Egypt; ^5^ Division of Biochemical Pharmacology, Department of Biology, University of Konstanz, Konstanz, Germany; ^6^ Department of Pharmacology and Biochemistry, Faculty of Pharmacy, Delta University for Science and Technology, Gamasa, Egypt

**Keywords:** alpelisib, phosphatidylinositol 3-kinase, acetaminophen, hepatotoxicity, DAMPs

## Abstract

The sterile inflammatory response mediated by Toll-like receptors (TLRs) 4 and 9 is implicated in the massive hepatic damage caused by acetaminophen (APAP)-overdose. There is a crosstalk between TLR-dependent signaling with other intracellular kinases like phosphatidylinositol 3-kinases (PI3Ks). Nevertheless, the detailed role of PI3Kα is still unknown in hepatic sterile inflammation. Accordingly, the effect of the novel PI3Kα inhibitor alpelisib was investigated in the setting of APAP-driven sterile inflammation in the liver. This was examined by pretreating mice with alpelisib (5 and 10 mg/kg, oral) 2 h before APAP (500 mg/kg, i.p.)-intoxication. The results indicated that alpelisib dose-dependently lowered APAP-induced escalation in serum liver function biomarkers and hepatic necroinflammation score. Alpelisib also attenuated APAP-induced rise in cleaved caspase 3 and proliferating cell nuclear antigen (PCNA) in the liver hepatocytes, as indices for apoptosis and proliferation. Mechanistically, inhibition of PI3Kα by alpelisib limited APAP-induced overproduction of the pro-inflammatory tumor necrosis factor (TNF)-α, interleukin (IL)-1β and IL-6 in the blood circulation *via* switching off the activation of several signal transduction proteins, including extracellular signal-regulated kinase (ERK), c-Jun N-terminal kinase (JNK), signal transducer and activator of transcription-3 (Stat-3), glycogen Synthase Kinase (GSK)-3β and nuclear factor (NF)-κB. Alpelisib also impaired APAP-instigated immune cell infiltration in the liver *via* reducing systemic granulocyte/macrophage-colony stimulating factor (GM-CSF) release and reversed APAP-induced abnormalities in the systemic and hepatic levels of the anti-inflammatory IL-10 and IL-22. In conclusion, selective modulation of the PI3Kα activity by alpelisib can hinder the inflammatory response and infiltration of immune cells occurring by APAP-hepatotoxicity.

## 1 Introduction

Acetaminophen (APAP) accounts for a high rate of mortality worldwide because of the massive hepatic failure caused by overdose. Hepatic inflammation is the most common pathological pattern distinguishing APAP-induced liver injury (Chang and Schiano, 2007). The sterile inflammatory response plays a major role in aggravating the first wave of hepatocellular damage initiated by N-acetyl-p-benzoquinone imine (NAPQI)-mediated depletion of hepatic GSH and the ensuing oxidative stress (Jaeschke and Ramachandran, 2020). This response is mainly driven by damage-associated molecular patterns (DAMPs) like high-mobility group B1 (HMGB1) and host DNA released from dying hepatocytes that can activate Toll-like receptors (TLRs) 4 and 9 localized on resident and recruited immune inflammatory (Shaker, 2022). Stimulation of these types of pattern recognition receptors (PRRs) leads to nuclear translocation of nuclear factor (NF)-κB and overproduction of pro-inflammatory cytokines like tumor necrosis factor (TNF)-α, interleukin (IL)-1β and IL-6 (Shaker et al., 2016). Hence, therapeutic strategies that interfered with either DAMPs, PPRs or their downstream successfully spared the liver from the inflammation cascade caused by APAP-overdose.

PRRs mediate the overexpression of pro-inflammatory cytokines *via* certain signal transduction proteins that are activated by certain kinases (Troutman et al., 2012). Of these kinases, phosphatidylinositol 3-kinases (PI3Ks) are intracellular lipid kinases that transduce signals for proliferation, survival, differentiation and metabolism (Pourrajab et al., 2015). While there are 3 classes of PI3Ks (I, II and III), Class IA PI3Ks are heterodimers comprising catalytic (p110) and regulatory (p85) subunits. PI3Ks are activated by receptor tyrosine kinases leading to biosynthesis of phosphatidylinositol-3, 4, 5-triphosphate (PIP3) and subsequent activation of protein kinase B (Akt) downstream signals (Jean and Kiger, 2014). Class IA isoforms like PI3Kα and PI3Kβ are ubiquitously expressed in all cells and tissues, while PI3Kδ is mainly enriched in leukocytes. Class IA PI3Ks are activated by tyrosine kinase receptors like those for the epidermal growth factor and insulin, as well as G-protein coupled receptors (Vanhaesebroeck et al., 2001). Class IB PI3Ks are composed of a catalytic subunit (p110γ) and a regulatory subunit (p101). PI3Kγ, belongs to class IB, plays a role in lymphocyte activation and inflammation following stimulation of G-protein coupled receptors and some tyrosine kinase receptors (Yang et al., 2019a).

Although several studies have identified crosstalk between TLRs and the PI3K/Akt signaling pathways, studies about the detailed role of PI3Kα in the liver are still limited and paradoxical. For instance, mice lacking the hepatic PI3K (p110α) were having impaired insulin sensitivity and glucose tolerance, as well as a rise in gluconeogenesis, hyperleptinemia and hypolipidemia (Sopasakis et al., 2010). Moreover, knockout mice for hepatic PI3K p110-α, but not p110-β, had lower magnitude of liver steatosis induced by high fat diet in comparison to wild type counterparts (Chattopadhyay et al., 2011). This effect was linked to PI3K p110-α role in regulating the atypical protein kinase C activation, lipogenesis and fatty acid incorporation into triglycerides in liver hepatocytes. In the context of liver fibrosis, selective inhibition of PI3K signaling in hepatic stellate cells reduced their ability to proliferate, migrate and produce collagen (Reif et al., 2003; Son et al., 2009). Pharmacological inhibition of the PI3K by omipalisib reduced fibrogenesis response of hepatic stellate cells alongside steatosis in precision-cut liver slices of a choline deficient L-amino acid fed mice (Gore et al., 2019). Otherwise, genetic ablation of p110-α subunit of PI3K in mice embryonic fibroblasts prevented the oncogenic transformation elicited by oncogenic receptor tyrosine kinases (Zhao et al., 2006). In a mice model of hepatectomy, selective inhibition of PI3K (p110α) with siRNA reduced hepatocyte proliferation, infiltration of macrophages and activation of Kupffer cells (Jackson et al., 2008).

Inhibition PI3K/Akt/mTOR signaling pathway can promote autophagy and decrease lipid deposition and inflammatory cytokines release in a high-fat diet mice model (Sun et al., 2023). PI3K can prohibit NF-κB activation and TLR4 signaling in monocytes *via* interfering with the recruitment of Toll-IL-1 resistance domain junction protein (TIR) toward the cellular membrane (Guha and Mackman, 2002; Acosta-Martinez and Cabail, 2022). On the other hand, inflammation responses caused by TLR9 stimulation to neutrophils disappeared by administration of PI3K inhibitors independent of the myeloid differentiation primary response 88 (MyD88)/IL-1 receptor-associated kinase 4 (IRAK-4)/inhibitor of NF-κB kinase (IKK)/NF-κB pathway (Hoarau et al., 2007). Moreover, aberrant activation of the PI3K(p110α) has been convicted for the progression of liver cancer (Li et al., 2015).

Alpelisib is an oral inhibitor of the class I PI3K that selectively inhibits the catalytic subunit p110α (Royer et al., 2023). Alpelisib was approved by Food and Drug Administration as a treatment for metastatic breast cancer and the manifestation of PIK3CA-related overgrowth spectrum in 2019 and 2022, respectively (André et al., 2019; Garreta Fontelles et al., 2022). Data about the role of PI3Kα in the context of APAP-driven sterile inflammation in the liver are still unavailable so far. In the current study, the impact of the pharmacological inhibition of PI3Kα by alpelisib was examined on APAP-induced injury, inflammation and regeneration in the liver.

## 2 Materials and methods

### 2.1 Animals

Male BALB/c mice (aged 11–12 weeks, weighing 28–32 g) were provided with food-pellets and water bottles 1 week during the adaptation prior to the experiments. Animal care and experimentation were consistent with the regulations of the National Institutes of Health and the institutional bioethical committees (Mansoura University-Faculty of Pharmacy, Code:2021-259).

### 2.2 Design of experiments and treatments

Alpelisib (formerly BYL719; Novartis, Switzerland) was dissolved in a solution of physiological saline containing carboxymethylcellulose 0.5% w/v to yield a concentration of 5 or 10 mg/10 mL, while APAP (Sigma-Aldrich, United States) was dissolved in a warm physiological saline as 500 mg/20 mL.

#### 2.2.1 Alpelisib pretreatment for APAP-intoxicated mice

Alpelisib treatments (5 and 10 mg/kg; 0.3 mL/30 g mice) were given orally 2 h before the intraperitoneal (i.p.) administration of APAP (500 mg/kg; 0.6 mL/30 g mice). APAP-dose was based on inducing a significant hepatic inflammation with least mortality (Shaker, 2014). The doses of alpelisib (5–10 mg/kg) were chosen to fulfill the efficacy and safety in mice and the clinical applicability (Gobin et al., 2015). The mice were assigned into the following 5 groups after overnight fasting:

(1) Control: Mice were administered the vehicle without alpelisib (0.3 mL/30 g mice, oral) and after 2h, received saline without APAP (0.6 mL/30 g mice, i.p.);
(2) APAP: Mice were administered the vehicle without alpelisib (0.3 mL/30 g mice, oral) and after 2h, received APAP (500 mg/kg);
(3) Control + Alpelisib (10 mg/kg): Mice were administered alpelisib (10 mg/kg) and after 2h, received saline without APAP (0.6 mL/30 g mice, i.p.);
(4) APAP + Alpelisib (5 mg/kg): Mice were administered alpelisib (5 mg/kg) and after 2h, received APAP (500 mg/kg); and
(5) APAP + Alpelisib (10 mg/kg): Mice were administered alpelisib (10 mg/kg) and after 2h, received APAP (500 mg/kg).

#### 2.2.2 Alpelisib treatment after APAP-intoxication

One group of mice received APAP as above and received saline without alpelisib (0.3 mL/30 g mice, oral) 2 h afterwards. Another group of mice received APAP as above and received saline containing N-acetylcysteine (NAC, Sigma, United States; 100 mg/kg/10 mL, 0.3 mL/30 g, oral) 2 h afterwards. The other 2 groups of mice were administered APAP as above and after 2 h or 4 h received saline containing alpelisib (10 mg/kg, oral).

### 2.3 Collection of samples

After 1, 4 or 24 h from APAP-challenge, the animals were euthanatized by thiopental (100 mg/kg/10 mL, 0.3 mL/30 g mice, i.p). After cardiac puncture for blood collection, blood samples were permitted to clot and centrifuged (3,000 *g*, 10 min, room temperature) for separation of serum. Thereafter, sera were kept at −20 °C for the latter biochemical evaluation of liver injury and ELISA. Liver samples of the 4 h APAP-challenge groups were collected and stored at −80 °C for Western blotting. Meanwhile, some of liver samples of 24 h APAP-challenge groups were either stored at −80 °C ELISA or fixed in 10% v/v of formalin in saline solution for the latter histopathological and immunostaining evaluations.

### 2.4 Biochemical evaluation of hepatic injury

Biochemical kits (Biomed diagnostics, Egypt) were employed to assess alanine transaminase (ALT), aspartate transaminase (AST) and lactate dehydrogenase (LDH) in serum samples.

### 2.5 Hepatic histopathological and immunostaining evaluations

Liver slices were fixed inside paraffin blocks, followed by cutting into 5 μm sections, displacement on glass slides and staining with hematoxylin and eosin (HE). Hepatocellular necroinflammation was assessed based on the following scores: 0, absent; 1 = spotty necrosis; few necrotic hepatocytes; 2 = confluent necrosis; and 3 = bridging necrosis (González-Périz et al., 2006).

Some sections were also displaced on coated slides for the immunostaining protocol with the primary antibodies. These include cleaved caspase 3 (Santa Cruz, United States), F4/80, Ly6G, proliferating cell nuclear antigen (PCNA) and NF-κB (BioLegend, United States). Thereafter, the sections were subjected to HRP-linked secondary antibody and visualized with a solution composed of 3,3′-diaminobenzidine and peroxide substrate. Immunostaining expression was determined by ImageJ software (United States).

### 2.6 Evaluation of the pro-inflammatory and anti-inflammatory cytokines

TNF-α, IL-1β, IL-6, IL-22 and granulocyte/macrophage colony-stimulating factor (GM-CSF) concentrations were determined in serum aliquots, while those of IL-10 and IL-22 were determined in serum aliquots and hepatic lysates. Hepatic IL-17A concentration was also quantified. The concentrations of these cytokines were determined by ELISA kits (MAX™ Deluxe Sets**,** BioLegend, United States). Briefly, portions from the liver (10% w/v) were homogenized and lysed in an ice cooled lysis buffer (10 mM Tris pH 7.4, 150 mM NaCl, 0.5% Triton X-100) containing a protease inhibitor cocktail (Roche Diagnostics, Germany). Thereafter, the lysed liver aliquots were centrifuged at (5,000 *g*, 10 min, 4 °C), and the clear supernatants were loaded on a 96-well plate precoated overnight with the appropriate capture antibody. Serum aliquots were prediluted in physiological saline (2:1), followed by loading on the 96-well plate like those of lysates. The routine protocol steps of ELISA were then followed as provided by the manufacturer. The concentration of protein was also assessed in the liver lysates by the Bradford’s assay (Bradford, 1976).

### 2.7 Western blotting of liver samples

Liver samples (20 mg of tissue/sample) were lysed and homogenized in 0.3 mL of an ice-cold RIPA buffer containing protease and phosphatase inhibitors (Sigma, United States). Then, liver samples were kept on ice for 20 min, followed by centrifugation (8,000 g, 20 min, 4 °C) and isolation of supernatants. Thereafter, 30 μg of protein per supernatant was added to Laemmali’s buffer, denatured by heat and resolved by sodium dodecyl sulfate-polyacrylamide gel electrophoresis technique. The separated proteins on gel were transblotted to a nitrocellulose membrane by the aid of an electric current at 4 °C. After preincubation with a blocking buffer supplemented bovine serum albumin, the membrane was subjected overnight at 4 °C to a primary antibody for the phosphorylated forms of c-Jun N-terminal kinase (p-JNK) (Cell Signaling, United States, Cat no. 4668), extracellular signal-regulated kinase (p-ERK, Cat no. 4370) (Cell Signaling, United States), signal transducer and activator of transcription-3 (p-Stat-3; Cat no. 9145) (Cell Signaling, United States), inhibitor of NF-κB (p-IκBα) (Abcam, United States; Cat no. ab133462), or glycogen Synthase Kinase (p-GSK)-3β at serine 9 position (Elabscience, United States; Cat no. E-AB-20886). Also, the membrane was incubated after stripping with β-actin (BioLegend, United States; Cat no. 664803) as a loading control. After numerous washing cycles, the primary antibodies attached to the membrane were then linked to a horseradish peroxidase-labeled secondary antibody (Cell Signaling, United States; Cat no. 7074) raised against the host of the primary antibody. Thereafter, the target proteins were detected by a chemiluminescent camera system in the presence of a chemiluminescent substrate, and the density of detected bands were quantitated by ImageJ software (United States).

### 2.8 Evaluation of hepatic GSH concentration

Liver samples of the control, APAP and APAP + Alpelisib (10 mg/kg) groups were also collected after 1 h from APAP-intoxication. Thereafter, liver samples were homogenized as 10% w/v in phosphate-buffered saline, followed by centrifugation (4,000 g, 10 min, 4 °C) and isolation of supernatants. Samples of hepatic supernatants (0.45 mL) were added to 0.05 mL of 50% (w/v) trichloroacetic acid for precipitation of proteins, followed by centrifugation (4,000 *g*, 10 min, 4 °C) and isolation of supernatants. Then, 0.2 mL of the isolated supernatant was diluted with 1 mL of 0.2 M Tris-HCl (supplied with 1 mM EDTA, pH 8.9), followed by addition of 0.05 mL methanol containing 10 mM 5,5′-dithiobis(2-nitrobenzoic acid) (Sigma, United States) for yellow color formation. The absorbances of samples were measured spectrophotometrically under similar conditions of a standard curve of GSH (Acros Organics, United States) at a wavelength of 412 nm (Moron et al., 1979).

### 2.9 Statistical analysis

The one-way ANOVA succeeded by Tukey-Kramer test was employed for analyzing the parametric data (means ± SE), whereas the Kruskal–Wallis ensued by Dunn’s test was applied for the non-parametric data of histopathological scoring. Normal distribution of data was determined using Shapiro-Wilk test. The GraphPad Prism 8 (United States) was used in statistical analysis. The level of statistical significance was set at a probability less than 0.05.

## 3 Results

### 3.1 Inhibition of PI3K by alpelisib limits APAP-induced elevation in biochemical and pathological parameters of hepatocellular injury

**FIGURE 1:**
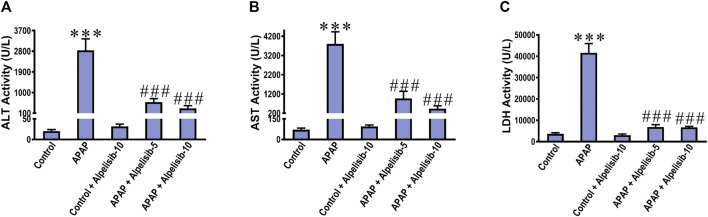
Impact of pretreatment with the PI3Kα inhibitor alpelisib (5 and 10 mg/kg) on APAP-induced alterations in activities of ALT **(A)** AST **(B)** and LDH **(C)** in sera after 24 h. Statistical significances (*n* = 6–7 per group) are denoted as ***p < 0.001 from the control group, while ###*p* < 0.001 from the APAP group.

**FIGURE 2:**
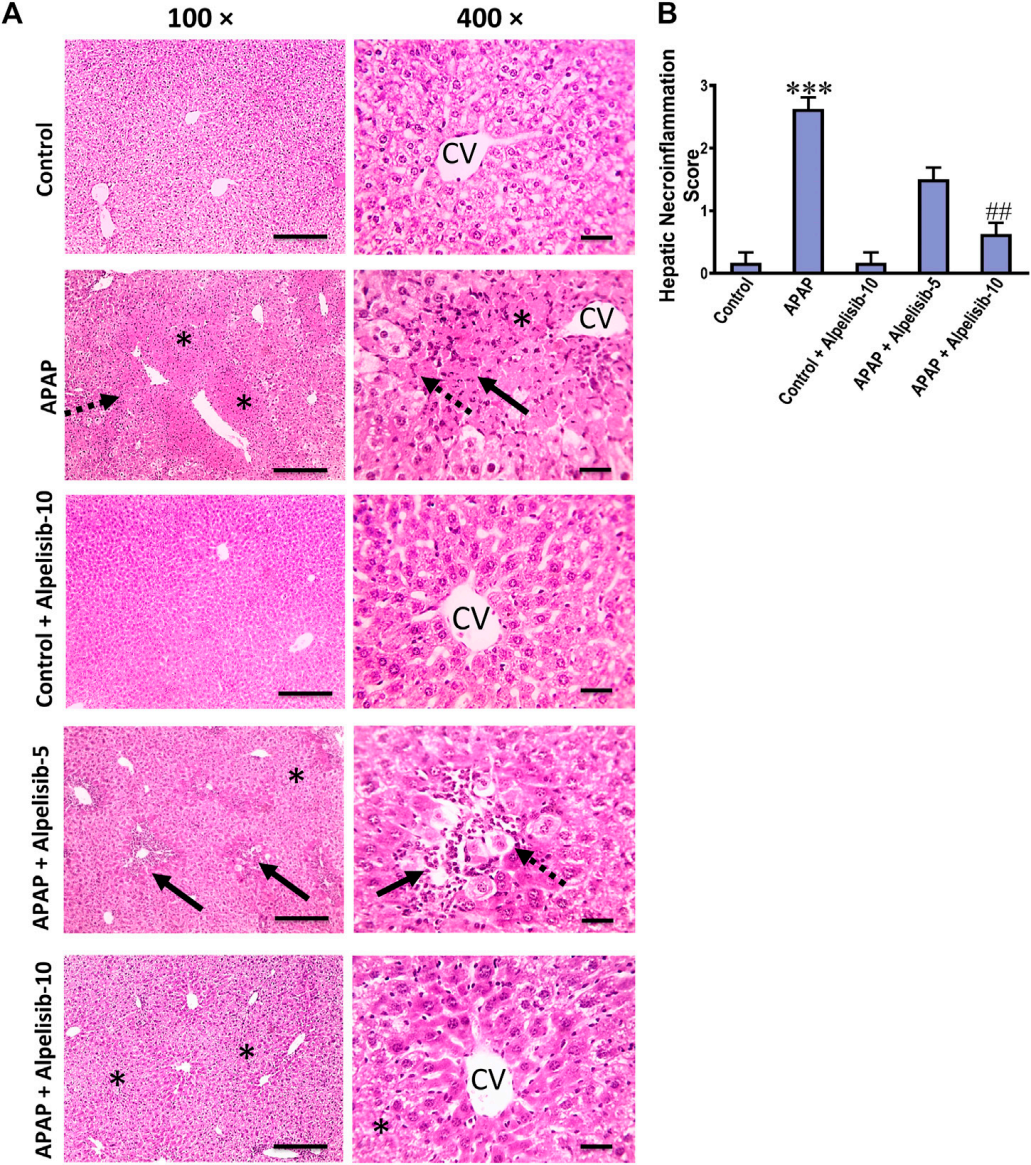
Impact of pretreatment with alpelisib (5 and 10 mg/kg) on APAP-induced alterations in hepatic histology **(A)** and necroinflammation score **(B)** after 24 h. Hepatic sections (HE staining; magnification power at ×100 (bar = 100 µm) and 400× (bar = 50 µm)) of the APAP-group showed severe necroinflammatory changes characterized by confluent areas of coagulative necrosis (*), manifesting more eosinophilic cytoplasm, nuclear pyknosis, and severe polymorphnuclear cells infiltration (straight black arrows), followed by a zone of hepatocellular hydropic or ballooning degeneration (dashed black arrows). Hepatic sections from the group received APAP + Alpelisib (5 mg/kg) had decreased necroinflammatory changes that characterized by small multifocal centriboular areas of hepatocellular coagulative necrosis (*), degeneration (dashed black arrows), apoptosis, mild inflammation and polymorphnuclear cells infiltration (straight black arrows). Hepatic sections from group received APAP + Alpelisib (10 mg/kg) had improved histological picture with mid areas of hepatocellular degeneration (*). Statistical significances (*n* = 6–7 per group) are denoted as ***p < 0.001 from the control group, while ##*p* < 0.01 from the APAP group.

There was a pronounced rise of ALT, AST and LDH activities in the sera of mice challenged with an overdose of APAP in comparison to those of the normal counterparts at *p* < 0.001 (Figures 1A–C). Besides, APAP-overdose elicited massive areas of necrosis in the hepatic tissue and significantly (*p* < 0.001) raised the necroinflammation score 2.5 folds over that of the normal level (Figures 2A, B). Intriguingly, these biochemical and pathological abnormalities driven by APAP were dose-dependently mitigated by pretreatment with alpelisib (5 and 10 mg/kg). Moreover, APAP-induced hepatocellular necrosis was markedly abolished by alpelisib at the higher dose level. Of note, oral administration of alpelisib (10 mg/kg) alone to the normal mice had no effect on markers of hepatic injury.

### 3.2 Alpelisib abates APAP-induced apoptosis and hepatocellular proliferation

**FIGURE 3:**
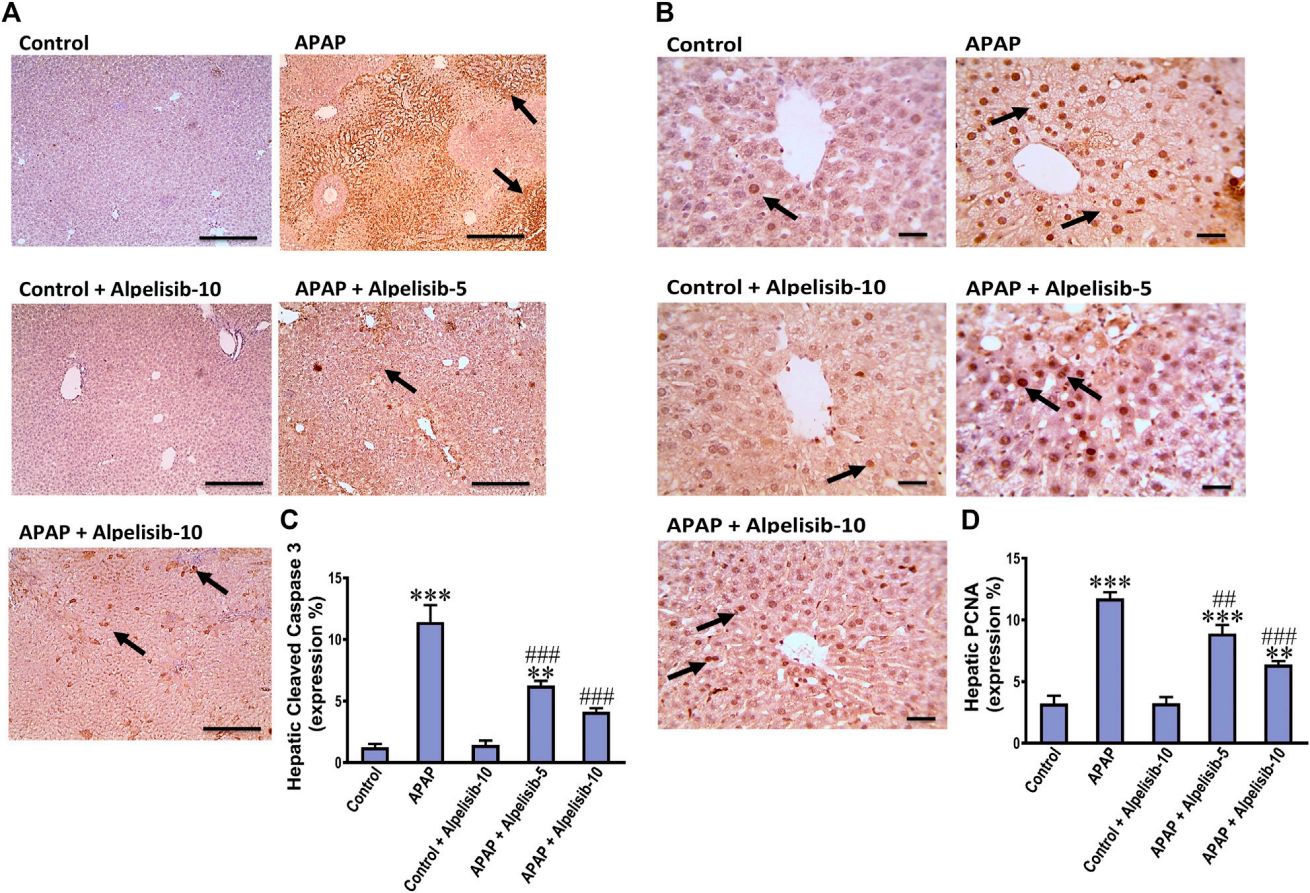
Impact of pretreatment with alpelisib (5 and 10 mg/kg) on APAP-induced alterations in immunostaining expressions of cleaved caspase 3 **(A,C)** and PCNA **(B,D)** in the liver after 24 h. Statistical significances (*n* = 6–7 per group) are denoted as *p < 0.05, **p < 0.01 and ***p < 0.001 from the control group, while ##*p* < 0.01 and ###*p* < 0.001 from the APAP group.

APAP-overdose may occasionally cause hepatocellular death by apoptosis if necrosis is inhibited. Thereby, we assessed the immunostaining of cleaved caspase 3 to investigate whether alpelisib can abate or aggravate apoptosis caused by APAP-overdose. The immunostaining results revealed that APAP-overdose increased the hepatic expression of cleaved caspase-3 (*p* < 0.001), compared to that of normal mice (Figures 3A, C). Meanwhile, alpelisib pretreatments (5 and 10 mg/kg) prior to APAP-challenge dose-dependently lowered the activity of this executioner caspase in the liver. Next, hepatocellular proliferation is known to occur after APAP-damage as an attempt for hepatocellular regeneration. Accordingly, we assessed the immunostaining of nuclear PCNA to reveal the impact of alpelisib on APAP-mediated hepatocellular regeneration. The immunostaining results revealed that APAP-hepatocellular damage was ensued with numerous positive nuclei staining for PCNA (*p* < 0.001) higher than the normal extent in control mice (Figurse 3B, D). Interestingly, this higher PCNA nuclear expression was lowered by both doses of alpelisib (5 and 10 mg/kg) pretreatments.

### 3.3 Inhibition of PI3K by alpelisib counteracts APAP-induced overproduction of the inflammatory IL-1β, TNF-α, and IL-6 in the blood circulation and IL-17A in the liver.

**FIGURE 4:**
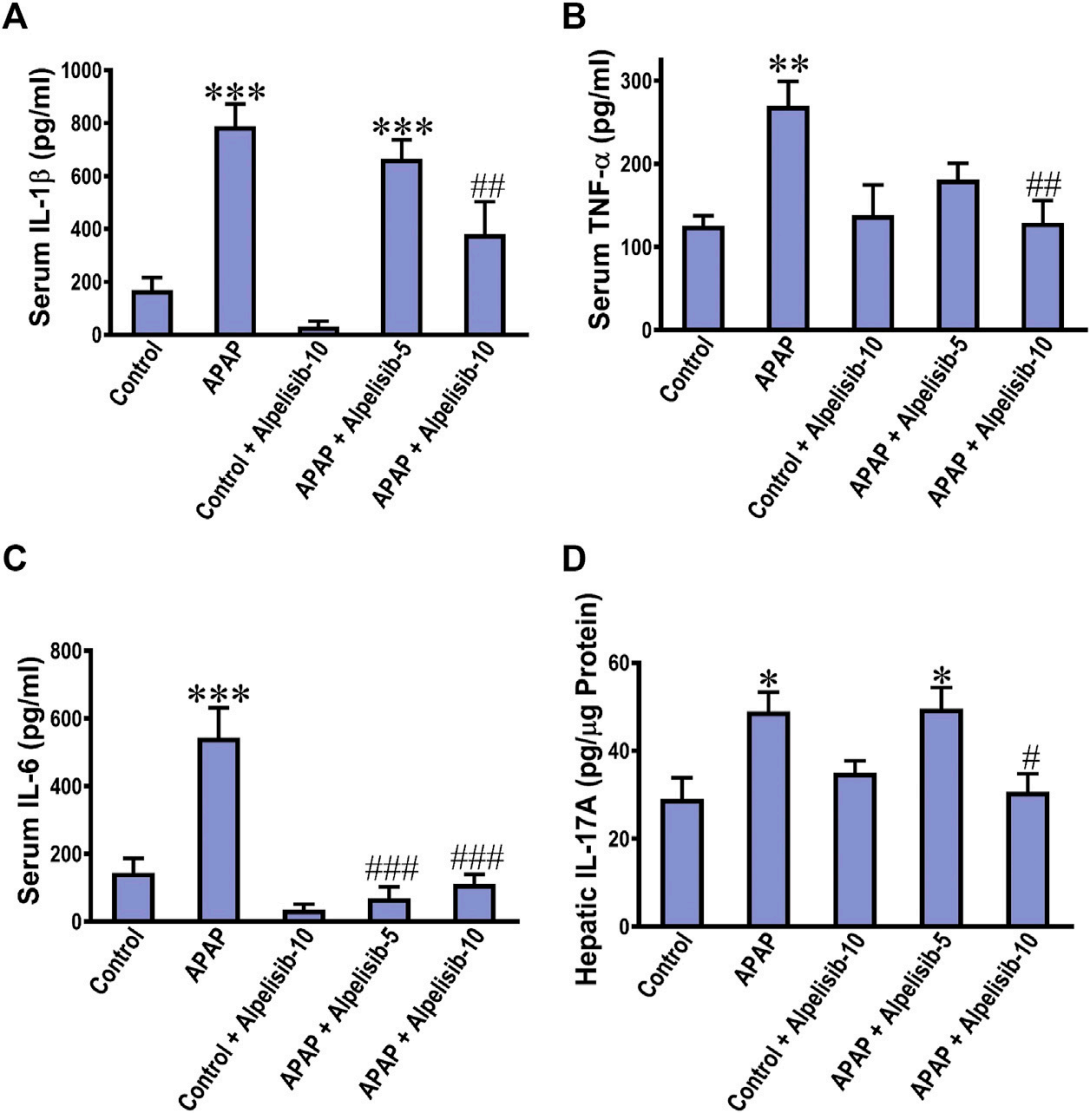
Impact of pretreatment with alpelisib (5 and 10 mg/kg) on APAP-induced alterations in concentrations of IL-1β **(A)**, TNF-α **(B)**, IL-6 **(C)** in sera and IL-17A in the liver **(D)** after 24 h. Statistical significances (*n* = 6–7 per group) are denoted as *p < 0.05, **p < 0.01 and ***p < 0.001 from the control group, while #*p* < 0.05, ##*p* < 0.01 and ###*p* < 0.001 from the APAP group.

The translocation of NF-κB toward the nucleus of inflammatory immune cells, either recruited or resident, drives the upregulation along with exogenous release of IL-1β, TNF-α and IL-6. Thereby, the concentrations of these cytokines were assessed in the systemic circulation by ELISA. The results indicated that the IL-1β, TNF-α, and IL-6 concentrations were elevated in serum samples of APAP-intoxicated mice in comparison to those of the normal counterparts (Figures 4A–C). Moreover, there was an increase in the hepatic expression of IL-17A due to APAP-intoxication (Figure 4D). Interestingly, alpelisib pretreatment, particularly at a dose of 10 mg/kg, countered APAP-elicited escalation of these inflammatory cytokines.

### 3.4 Inhibition of PI3K by alpelisib hinders APAP-induced dysregulation of the anti-inflammatory IL-10 and IL-22 in the blood circulation and liver

**FIGURE 5:**
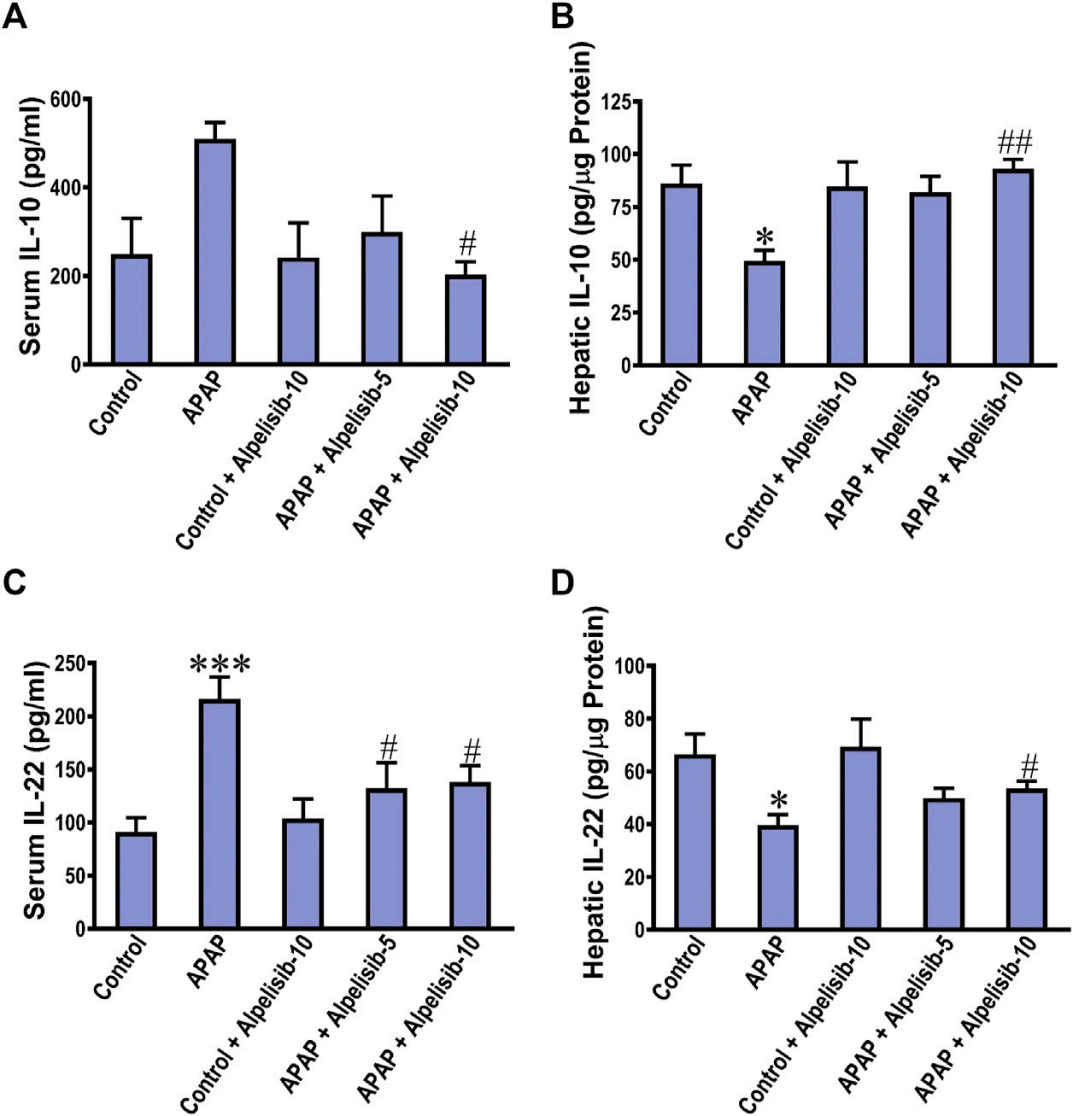
Impact of pretreatment with alpelisib (5 and 10 mg/kg) on APAP-induced alterations in concentrations of IL-10 **(A,B)** and IL-22 **(C,D)** in the serum and liver samples after 24 h. Statistical significances (*n* = 6–7 per group) are denoted as *p < 0.05 and ***p < 0.001 from the control group, while #*p* < 0.05 and ##*p* < 0.01 from the APAP group.

The anti-inflammatory response is driven by cytokines like IL-10 and IL-22 as an attempt to lower the inflammation and induce repair. Next, we investigated the impact of APAP alone or with alpelisib on the systemic and hepatic levels of the anti-inflammatory IL-10 and IL-22. APAP-insult was associated with a marked increase in the concentrations of these cytokines in serum samples, but a decline in the hepatic counterparts (Figures 5A–D). At the dose of 10 mg/kg, alpelisib successfully reversed APAP-elicited abnormalities in the systemic and hepatic levels of IL-10 and IL-22.

### 3.5 Alpelisib mitigates APAP-induced phosphorylation of ERK, JNK, Stat3, GSK-3β and IκB

**FIGURE 6:**
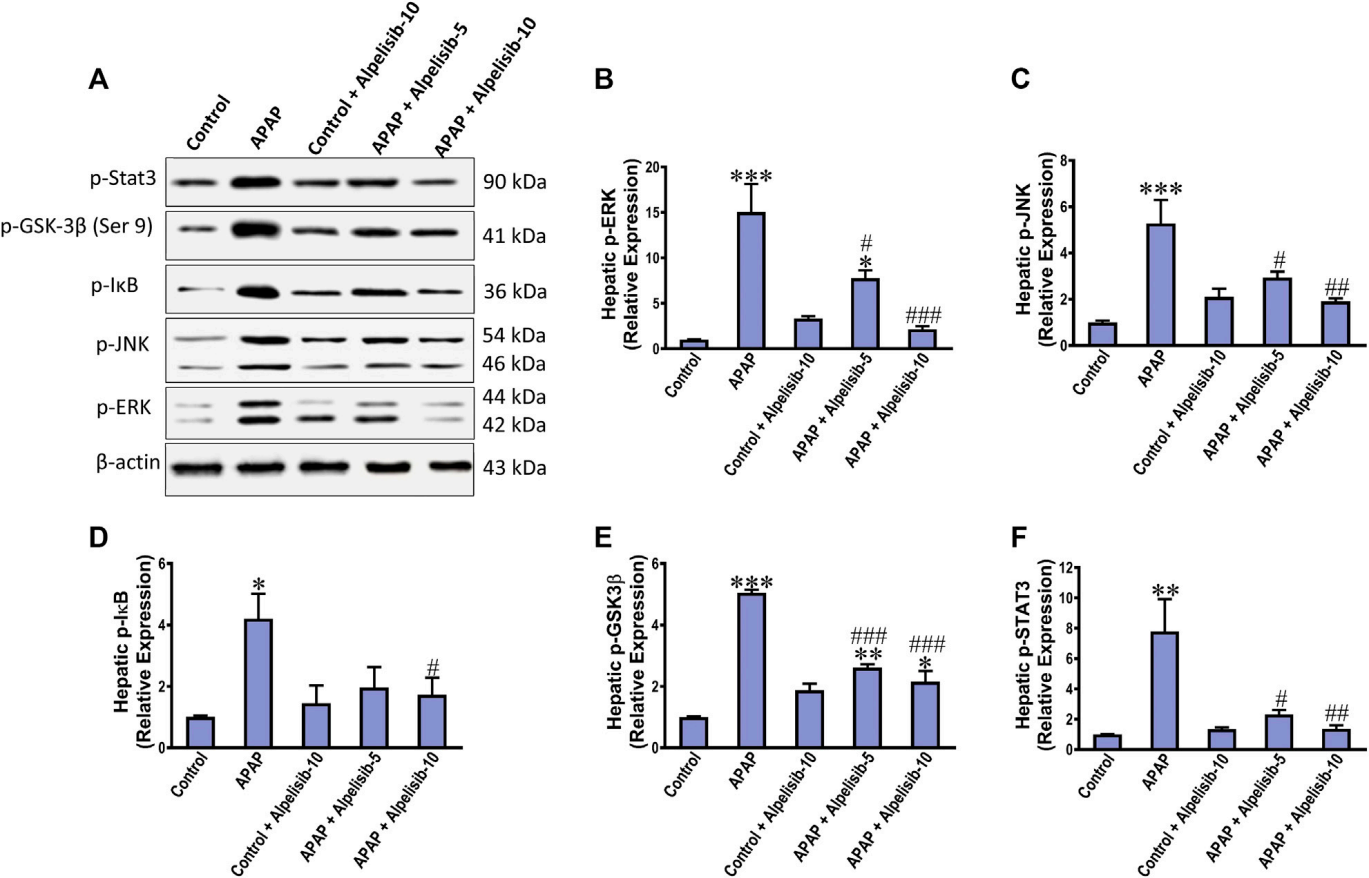
Impact of pretreatment with alpelisib (5 and 10 mg/kg) on APAP-induced alterations in hepatic phosphorylation of Stat 3, JNK, ERK, GSK3B and IκB after 4 h as determined by Western blotting **(A)** and the densitometric analysis **(B–F)**. Statistical significances (*n* = 3 per group) are denoted as *p < 0.05, **p < 0.01 and ***p < 0.001 from the control group, while #*p* < 0.05, ##*p* < 0.01 and ###*p* < 0.001 from the APAP group.

**FIGURE 7:**
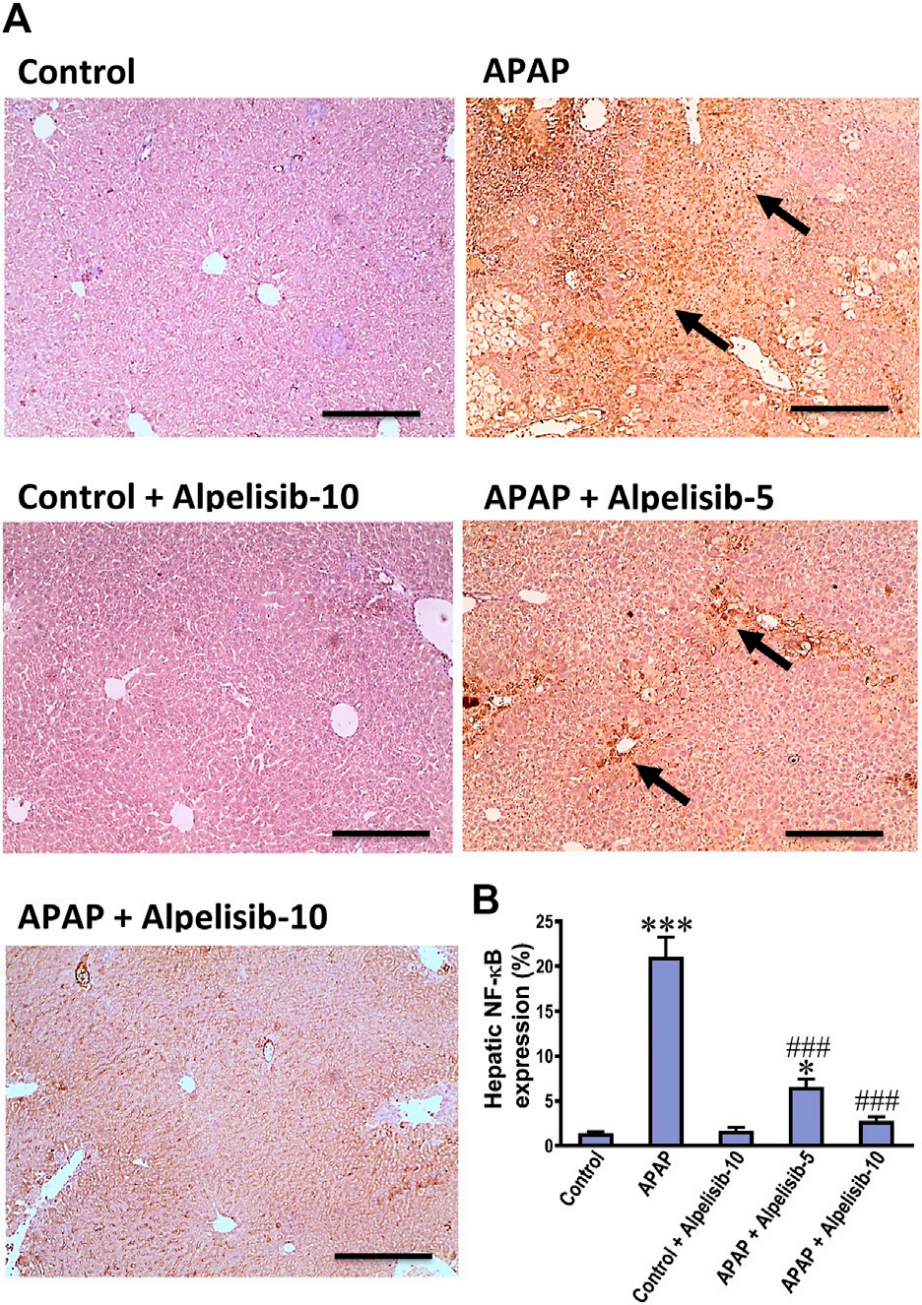
Impact of pretreatment with alpelisib (5 and 10 mg/kg) on APAP-induced alterations in immunostaining expressions of NF-κB **(A,B)** in the liver after 24 h. Statistical significances (*n* = 6–7 per group) are denoted as *p < 0.05 and ***p < 0.001 from the control group, while ###*p* < 0.001 from the APAP group.

As an attempt to identify the potential downstream signals controlled by PI3K, we assessed the hepatic phosphorylation of ERK, JNK, Stat3, GSK-3β and IκB after 4 h from APAP-challenge. The Western blotting results indicated that there were pronounced elevations in the hepatic phosphorylation of ERK (15-fold), JNK (5.3-fold), Stat3 (7.8-fold), GSK-3β (5.1-fold) and IκB (4.2-fold) due to challenging the mice with APAP (Figures 6A–F). However, these elevations were significantly reduced to the normal control mice levels upon pretreating the mice with alpelisib (10 mg/kg) prior to APAP. Following phosphorylation of IκB, the cytoplasmic NF-κB moves to the nucleus for gene transcription of mediators related to inflammation, apoptosis or regeneration. Consistently, the immunostaining results revealed that APAP-induced rise in the hepatic NF-κB expression was dose-dependently decreased by alpelisib pretreatments (Figures 7A, B).

### 3.6 Alpelisib interferes with APAP-induced recruitment for inflammatory cells in the liver via reducing GM-CSF

**FIGURE 8:**
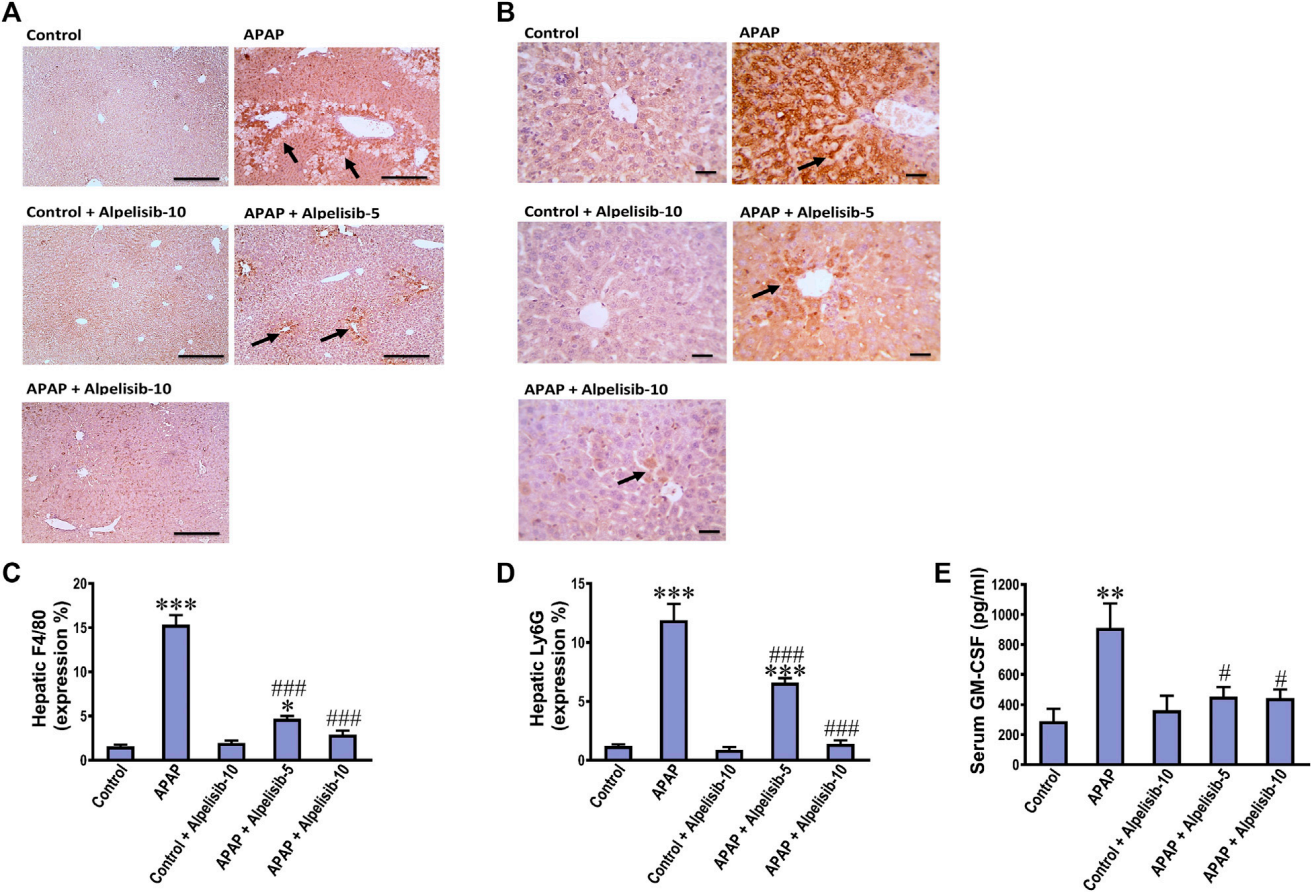
Impact of pretreatment with alpelisib (5 and 10 mg/kg) on APAP-induced alterations in immunostaining expressions of F4/80 **(A,C)** and Ly6g **(B,D)** in the liver and concentration of GM-CSF **(E)** in serum after 24 h. Statistical significances (*n* = 6–7 per group) are denoted as *p < 0.05, **p < 0.01 and ***p < 0.001 from the control group, while #*p* < 0.05 and ###*p* < 0.001 from the APAP group.

Inflammatory immune cells like monocytes and neutrophils transfer from the blood circulation toward the site of hepatic damage to aggravate the initial insult elicited by APAP. According, F4/80 and Ly6G-positive cells were assessed by immunostaining as indices for monocytes and neutrophils, respectively. APAP-intoxication elicited a marked (*p* < 0.001) increase in the expression of F4/80 (Figures 8A, C) and Ly6G-positive cells (Figures 8B, D) in the liver, compared to sections from the normal controls. The white blood cell growth factor GM-CSF drives stem cell conversion to granulocytes (neutrophils, basophils and eosinophils) and monocytes. Accordingly, the serum concentration of GM-CSF was determined to investigate the impact of APAP alone or when combined with alpelisib. As expected, challenge mice with APAP caused a pronounced (*p* < 0.01) rise in serum GM-CSF concentration, relative to the concentration of the normal counterparts (Figure 8E). Meanwhile, pretreating the mice with alpelisib (5 or 10 mg/kg) substantially (*p* < 0.05) abated APAP-mediated rise in GM-CSF release, compared to APAP-untreated mice.

### 3.7 Alpelisib does not reverse APAP-induced depletion of GSH in the liver

**FIGURE 9:**
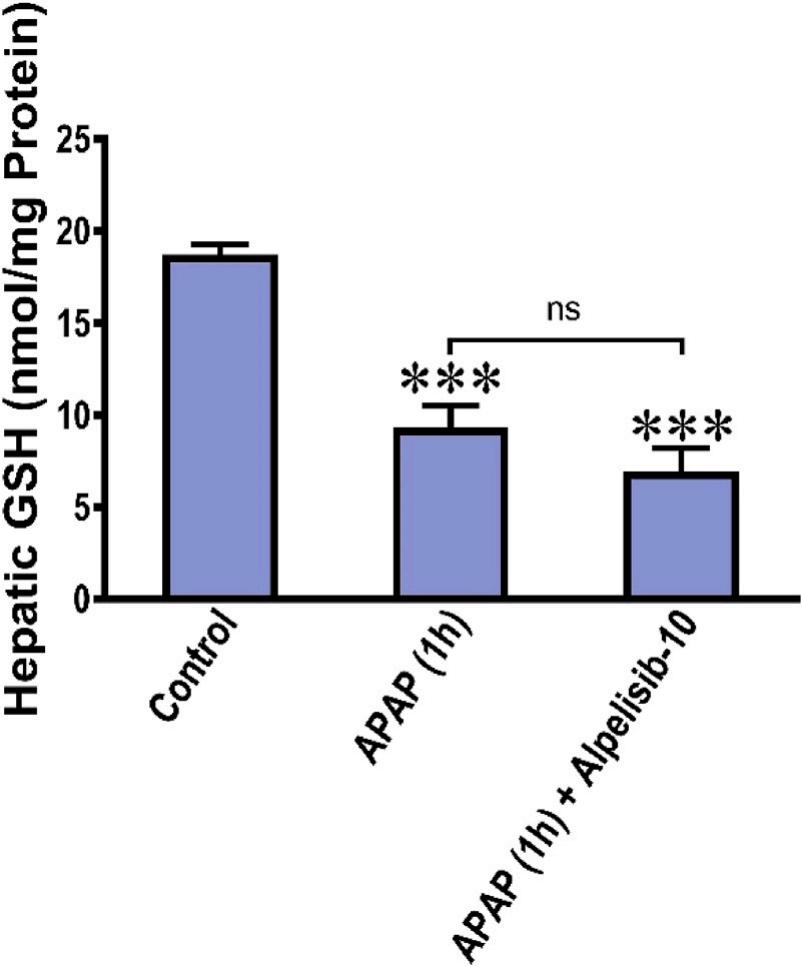
Impact of treatment with alpelisib (10 mg/kg) before 2 h from APAP-challenge and the alterations in concentrations of hepatic GSH after 1 h from APAP-challenge. Statistical significance is denoted as ***p < 0.001 from the control group.

APAP-overdose generates copious amount of NAPQI that depletes GSH and drives the initial wave of hepatocellular damage. Accordingly, we assessed the hepatic GSH concentration after 1 from APAP challenge with or without alpelisib to ascertain whether the hepatoprotective effect of alpelisib can be attributed to prevention of APAP-induced GSH depletion. The result indicated that pretreatment of alpelisib (10 mg/kg) could not reverse the decline in hepatic GSH concentration caused by APAP-overdose for 1 h (Figure 9).

### 3.8 Alpelisib can be used a treatment after APAP-challenge

**FIGURE 10:**
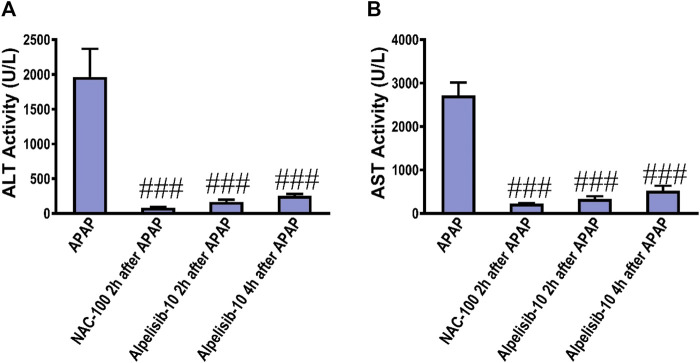
Impact of treatment with alpelisib (10 mg/kg) after 2 and 4 h from APAP-challenge and the associated changes in serum activities of ALT **(A)** and AST **(B)** after 24 h. Statistical significance is denoted as ###*p* < 0.001 from the APAP group.

To assess the possible clinical application of alpelisib for treatment of APAP-induced hepatotoxicity, alpelisib was given to mice as a treatment after 2h and 4 h from APAP-administration. Interestingly, alpelisib (10 mg/kg) could still limit APAP-elicited rise in serum ALT and AST activities to an extent comparable to NAC (100 mg/kg), especially when administered after 2 h from the intoxication (Figures 10A, B).

## 4 Discussion

Iatrogenic liver injury is linked to about 10% of acute hepatitis cases and represents an urgent cause for regulatory authorities to withdraw a pharmaceutical product from the market (O'Donnell et al., 2014). Because of the limited therapeutic options, investigating new agents that interfere with the subcellular inflammatory cascades would be of great importance for combating iatrogenic liver injury. Accordingly, we investigated the effect of the novel PI3Kα-specific inhibitor alpelisib on APAP-induced liver inflammation and the underlying mechanism of action behind such effect. The results indicated that the inhibition of PI3Kα by alpelisib ameliorated APAP-hepatotoxicity in mice as denoted by lowering the rise in serum liver function biomarkers (AST, ALT and LDH) and necroinflammation score (hepatic HE staining). The type of hepatocellular death in APAP-model is dominantly necrosis, but there may an occasional apoptosis secondary to the inhibition of APAP-necrosis (Du et al., 2019). The hepatic cleaved caspase 3 immunostaining revealed that alpelisib did not elicit apoptosis secondary to inhibition of APAP-necrosis.

Immune cell infiltration is intuitively responsible for the cytokine storm and a second much stronger wave of oxidative stress in the context of APAP-hepatotoxicity. As a next step, we investigated the ability of alpelisib to abrogate immune cell infiltration into the liver. Alpelisib prior to APAP-challenge lessened the recruitment of neutrophils (Ly6G) and monocytes (F4/80), as well as the systemic release of GM-CSF. GM-CSF acts on resident and/or migrated immune cells to promote their survival, maturation and trafficking of myeloid cells toward the injured tissue, as well as the overproduction of pro-inflammatory mediators (Hamilton, 2020).

Although neutrophils can participate in APAP-induced liver injury, they can also contribute to tissue repair and orchestrate resolution of inflammation. For instance, the depletion of neutrophils by anti-Gr-1 reduced the capability of production of ROS, leading to less serum ALT release and hepatocellular necrosis in APAP-challenged mice (Liu et al., 2006). Moreover, neutrophil depletion or abolishing neutrophil extracellular traps by DNase decreased the HMGB1 level and hindered hepatocellular necrosis (Liu et al., 2021). Meanwhile, recent advances indicated that early neutrophil depletion lessened hepatic injury, but late neutrophil depletion decreased the hepatocellular repair and proliferation (Guo et al., 2021; Yang et al., 2022). Although monocytes are recruited to aggravate the inflammatory response, they can mature into monocyte-derived macrophages with the aid of neutrophils to resolve the inflammation (Yang et al., 2019b). Moreover, neutrophil extracellular traps can be induced by stimulation of neutrophils with TNF-α or GM-CSF, and these responses were recently found to be dependent on activation of PI3Kα and PI3Kγ isoforms (de Carvalho Oliveira et al., 2023). Taken these findings together, PI3Kα inhibition by alpelisib countered APAP-mediated release of HMGB1 due to hepatic necrosis and interfered with TNF-α and GM-CSF-mediated release NETs from neutrophils.

Pooling of such immune cells in the inflamed hepatic tissue releases numerous arrays of cytokines. Among these, TNF-α has an axial role in maintaining liver homeostasis as it stimulates hepatocellular regeneration via DNA synthesis in normal hepatocytes. However, once exposed to a hepatotoxin, TNF-α signaling is transferred from proliferation to apoptosis by mediating crosstalk with heterologous signaling pathways. Such pathways include the canonical NF-κB activation, the stress-activated protein kinase JNK, and activation of the mitochondrial apoptotic pathway and proteolytic activation of the apoptosis executioner caspase-3 (Hayden and Ghosh, 2014; Liu et al., 2017). Interestingly, alpelisib (10 mg/kg) could suppress such inflammatory cascade as evidenced by reducing APAP-escalated release of TNF-α, phosphorylation of IκB, nuclear expression of p-65, phosphorylation of JNK and cleavage of caspase-3.

NF-κB upregulates the proinflammatory pro-IL-1β (Liu et al., 2017) that is implicated alongside IL-6 in driving the acute phase response (Bode et al., 2012). Meanwhile, alpelisib, especially the 10 mg/kg dose, was able to suppress APAP-instigated release of IL-1β and IL-6 in the systemic circulation that was concordant with lowering the activation of NF-κB alongside Stat3. Pro-IL-1β is fully processed by cleaved caspase 1 subsequent to P2X7 activation by ATP and NLRP3 inflammasome assembly (Hoque et al., 2012). Alpelisib mediated reduction of IL-1β release can be linked to suppression of PI3K/AKT pathway, which was recently found to promote the NLRP3-inflammasome activation in LPS-stimulated macrophages (Wang et al., 2020). Thus, PI3Kα inhibition seems to be an effective therapeutic strategy that could interfere with either DAMPs, PPRs or their downstream for sparing the liver from APAP insult. Otherwise, the exuberant IL-6-dependent signaling results in a sustained activation of Stat3 which in turn raises the IL-6 expression and release (Garbers et al., 2015). Hence, this positive feedback loop was abated by alpelisib as confirmed by reducing the systemic release of IL-6 and activation of Stat3 in the liver.

IL-17A, another contributor to the inflammatory status, is mainly produced by Th17 cells. IL-17A stimulation to IL-17R can activate NF-κB and MAPK leading to overproduction of TNF-α and IL-6 from Kupffer cells (Yan et al., 2012). Besides, IL-17A is capable of inducing GM-CSF production from stromal cells that drives neutrophils recruitment to the inflammation site (Iwakura and Ishigame, 2006; Jin and Dong, 2013). Hence, the 10 mg/kg dose of alpelisib was capable of reducing APAP-induced rise in the hepatic IL-17 content that also withholds the deleterious dependent effects in the liver.

Activation of PI3K mediates the generation of PIP3 and phosphorylation of Akt, which can subsequently phosphorylate several proteins including GSK-3β (Rayasam et al., 2009). While GSK-3β is functionally active in the cytosol, its activity is augmented or inhibited via phosphorylation at tyrosine 216 or serine 9 sites, respectively (Augello et al., 2020). Inactivation of GSK-3β drives the anti-inflammatory response *via* upregulating the anti-inflammatory IL-10 and modulating signaling inhibitors that can terminate the inflammatory pathways elicited by TLR4/NF-κB dependent signaling (Cortés-Vieyra et al., 2021). However, inhibition of GSK-3β was reported to increase hepatocyte apoptosis toward TNF-α (Schwabe and Brenner, 2002). Although both forms of GSK-3β (tyrosine 216 and serine 9) have been activated after 4 h from APAP-challenge in the mice hepatocytes, silencing or inhibition of GSK-3β attenuated JNK activation and mitochondrial dysfunction caused by APAP-overdose (Shinohara et al., 2010). Taking these findings together, alpelisib pretreatments reduced APAP-induced inhibitive phosphorylation of GSK-3β serine 9 in a dose-dependent manner, which indicates sparing of the liver cells from the deleterious abnormalities elicited by APAP-overdose.

The flood of pro-inflammatory cytokines serves as a protective attempt to remove the injurious stimuli. However, if not controlled, it would result in a potentially dangerous situation (Mauriz et al., 2013). Inflammation caused by APAP-challenge was associated with increased systemic release of the anti-inflammatory cytokines IL-10 and IL-22 levels to buffer inflammation, while their hepatic levels were decreased. IL-10 exhibits its anti-inflammatory effects through multiple effectors including NF-κB signaling inhibition and modulating Stat3 signaling, which has dual pro- and anti-inflammatory capabilities relying on the acting cytokine (O'Shea and Murray, 2008; Sanjabi et al., 2009). APAP-induced alterations in both hepatic and serum IL-10 concentrations were reversed by alpelisib, especially the 10 mg/kg dose.

IL-22 is distinguished from other cytokines in that it signals directly to of acting directly on the tissue that expresses functional IL-22R1 and does not serve to communicate between different leukocytes. Although IL-22 has a critical role in provoking acute phase response for maintaining tissue integrity during inflammation (Liang et al., 2010), it also has a key role in tissue recovery after injury through enhancing the proliferative and antiapoptotic programs (Chestovich et al., 2012). According to our results, APAP-induced increase in the release of IL-22 in the circulation was dampened by both doses of alpelisib. On the other side, the hepatic content of IL-22 was elevated by the 10 mg/kg dose of alpelisib, compared to the diseased group, indicating the improved regenerative capacity of hepatocytes after injury.

The impact of alpelisib was also elucidated on APAP-instigated hepatocellular regeneration by evaluating the nuclear expression of PCNA and the phosphorylation of ERK, a pro-proliferative component of the MAPK signaling pathway. While APAP escalated the nuclear staining of PCNA and hepatocellular ERK protein expression, alpelisib pretreatment successfully curbed these rises. These findings indicate that APAP-mediated regeneration after injury is dependent on the ERK phosphorylation, whereas pretreatment with alpelisib curbed both processes in the liver. Of note, alpelisib hepatoprotective effects mediated by PI3K inhibition appear to be primarily related to impairment of activation JNK and NF-κB in the period of 4 h from APAP-intoxication, rather than prevention of hepatic GSH. This finding was corroborated by maintenance of alpelisib beneficial effects when given after 2 h and 4 h from APAP-intoxication.

**FIGURE 11:**
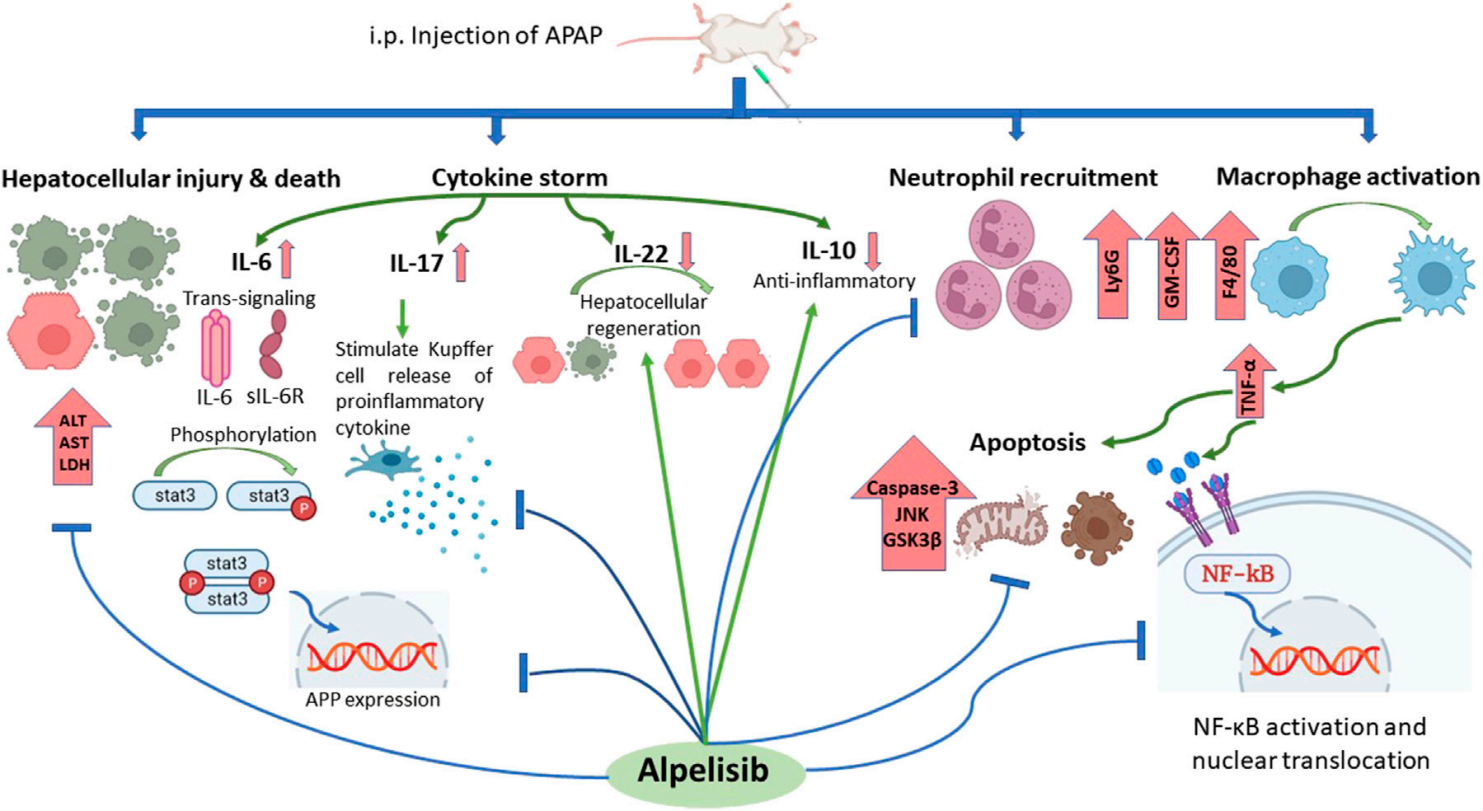
Summary of alpelisib actions on APAP-induced hepatoxicity.

In conclusion, inhibition of PI3Kα with alpelisib not only can prevent but also treat APAP-induced liver toxicity *via* reduction of immune cells infiltration and inflammatory cytokines production (Figure 11). Alpelisib may become a valuable candidate to treat this type of iatrogenic liver injury that is commonly encountered in humans.

## Data Availability

The original contributions presented in the study are included in the article/Supplementary Material, further inquiries can be directed to the corresponding author.
