# Corrigendum: Mitigation of acetaminophen-induced liver toxicity by the novel phosphatidylinositol 3-kinase inhibitor alpelisib

**DOI:** 10.3389/fphar.2023.1281416

**Published:** 2023-09-05

**Authors:** Mohamed E. Shaker, Hesham A. M. Gomaa, Sara H. Hazem, Mohamed A. Abdelgawad, Mohamed El-Mesery, Ahmed A. Shaaban

**Affiliations:** ^1^ Department of Pharmacology, College of Pharmacy, Jouf University, Sakaka, Al-Jouf, Saudi Arabia; ^2^ Department of Pharmacology and Toxicology, Faculty of Pharmacy, Mansoura University, Mansoura, Egypt; ^3^ Department of Pharmaceutical Chemistry, College of Pharmacy, Jouf University, Sakaka, Al-Jawf, Saudi Arabia; ^4^ Department of Biochemistry, Faculty of Pharmacy, Mansoura University, Mansoura, Egypt; ^5^ Division of Biochemical Pharmacology, Department of Biology, University of Konstanz, Konstanz, Germany; ^6^ Department of Pharmacology and Biochemistry, Faculty of Pharmacy, Delta University for Science and Technology, Gamasa, Egypt

**Keywords:** alpelisib, phosphatidylinositol 3-kinase, acetaminophen, hepatotoxicity, DAMPs

In the published article, there was an error in **Affiliation** for first author “Mohamed E. Shaker.” Instead of affiliations 1 and 2, the author should only have affiliation 1.

The authors apologize for this error and state that this does not change the scientific conclusions of the article in any way. The original article has been updated.

